# Phylogenomic investigation of safflower (*Carthamus tinctorius*) and related species using genotyping-by-sequencing (GBS)

**DOI:** 10.1038/s41598-023-33347-0

**Published:** 2023-04-17

**Authors:** Somayeh Sardouei-Nasab, Zahra Nemati, Ghasem Mohammadi-Nejad, Reza Haghi, Frank R. Blattner

**Affiliations:** 1 Leibniz Institute of Plant Genetics and Crop Plant Research (IPK), 06466 Gatersleben, Germany; 2 Research and Technology Institute of Plant Production (RTIPP), Shahid-Bahonar University of Kerman, P.O.B, 76169-133, Kerman, Iran

**Keywords:** Evolution, Plant sciences

## Abstract

Safflower (*Carthamus tinctorius*, Asteraceae) is a source of high-quality edible oil growing in moisture-limited environments. Despite its economic importance, the relationships to close wild species in *Carthamus* and the presence and relationships of ecotypes within safflower are still not fully clarified. Here we use genotyping-by-sequencing to identify the wild progenitor of *C. tinctorius*, infer phylogenetic relationship within the series *Carthamus* and identify groups of closely related lineages within cultivated safflower. Phylogenetic and population genomic analyses found *C. palaestinus* to be the closest relative and single progenitor of *C. tinctorius*, which confirms the Levant as the area of domestication of the crop. Flow cytometry showed all analyzed samples of *C. oxyacantha*, *C. palaestinus* and C. *tinctorius* to be diploid (2*n* = 2*x* = 24) with 2C genome sizes of 2.4–2.7 pg. Analyses of a set of 114 worldwide distributed safflower accessions arrived at two to five genetic groups, which showed, however, no correlation with the geographic origins of these accessions. From this, we conclude that the trade of safflower seeds resulted in multiple introductions of genotypes from the Levant into other areas with suitable climate conditions for the plant, as well as exchange of genotypes among these areas.

## Introduction

The genus *Carthamus* L. (Asteraceae, Carduoideae) consists of about 20 species with a distribution center in the Eastern Mediterranean and Irano-Turanian region^1,2^. Generic delimitation^3,4^ as well as intrageneric units are for a long time under debate. Hanelt^5^ divided *Carthamus* into five sections based on morphological characters, the same number was proposed by Estilai and Knowles^6^ based on cytological information. Vilatersana et al.^3,7^ using DNA marker regions divided the genus into two sections (section *Carthamus* and section *Atractylis* rchb.). This latter grouping was also followed by Bowles et al.^1^ and the split of *Carthamus* in two major clades was found multiple times with different molecular analysis methods [e.g., Refs.^8,9^] without formally referring to the sections. Section *Carthamus* species are assumed to be all diploid, while species within section *Atractylis* are polyploids. Unclear is the position of *C. arborescens* L.^1^ that sometimes is placed in the closely related genus *Phonus* hill^3,4^.

Safflower (*Carthamus tinctorius* L.), the only cultivated species of the genus, was domesticated in the Fertile Crescent over 4000 years ago^10,11^, most probably for its use as colorant. Nowadays it is mainly cultivated as an oilseed crop in suitable areas of Asia, northeastern Africa, Australia, the Americas and Europe^2,12,13^. With a total cultivation area of 1.1 million hectares a seed yield of approximately 1 million tons per year is reached^14^. Despite its growing commercial importance, genetic diversity and the parental origin of safflower are not fully clarified mainly due to limited taxon sampling among its close relatives included in phylogenetic studies, and partly also due to the low information content of fragment analyses for interspecific phylogenies such as RAPDs^2,7^, conserved intron-spanning PCR markers^12^, microsatellite markers^15^, ribosomal DNA RFLP analyses^2^, ISSR^16^, EST-SSR^17^ and combinations of these methods^2^. Some of the previous reports considered *C. oxyacantha* m.bieb., a species widespread in western Asia, as the wild progenitor of *C. tinctorius*^17–19^, while others found *C. palaestinus* eig from the Fertile Crescent the most likely progenitor^12,20,21^. The contribution of both *C. palaestinus* and *C. oxyacantha* in the origin of safflower has been reported based on chloroplast DNA diversity^22^. However, this latter study was based on limited taxon sampling, as just one individual from each of the wild species and two safflower individuals were included. In contrast to the phylogenetic analyses of diverse *Carthamus* species, within *C. tinctorius* analyses of length differences in PCR-amplified fragments mostly revealed high genetic diversity among safflower accessions^2,7,12,16,23–25^.

Here we use genome-wide single-nucleotide polymorphisms (SNP) obtained through genotyping-by-sequencing (GBS)^26^ on a diverse set of *C. tinctorius* accessions and six wild *Carthamus* species for a high-resolution phylogeny. The objectives of our study are (i) to infer phylogenetic relationships of *Carthamus* species closely related to safflower, i.e. section *Carthamus*, (ii) to confirm the closest wild relative(s) of safflower to enable breeding for the increase of genetic diversity regarding fatty acid content, drought tolerance and other agro-morphological traits, (iii) to look into genetic diversity of safflower accessions, and (iv) to see if the three to ten postulated morphology and geography-defined groups within *C. tinctorius*^23,25,27–31^ are genetically distinct and can be recovered using genomic data.

## Results

### GBS sequencing

After removing the individual sample barcodes and quality filtering, our GBS data resulted in on average 1582 loci (min: 1409, max: 1614) per individual with 61 × coverage (min: 31 ×) for a dataset with 60 individuals covering safflower plus six wild *Carthamus* species with 3.5% missing sites.

For a larger dataset consisting of all *C. tinctorius* individuals together with three closely related wild species belonging to section *Carthamus* we obtained on average 7197 loci (min: 4087, max: 7513) per individual with a coverage of 163 × (min: 40 ×) with overall 8.7% missing sites.

### Phylogenetic inference

**Figure 1:**
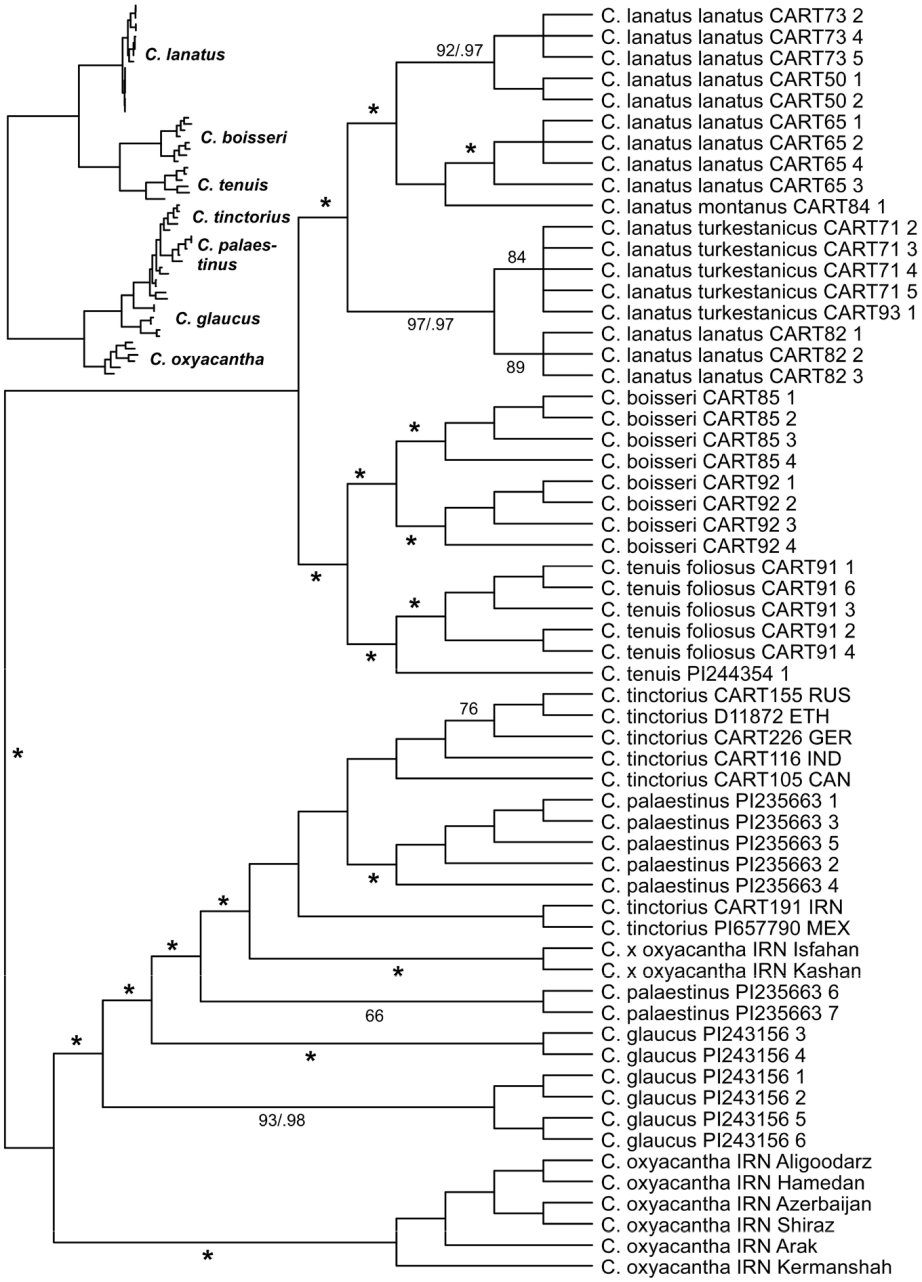
Unrooted strict consensus tree of 2484 MP trees derived from the analysis of GBS data. Asterisks indicate branches with support values of ≥ 98% in MP bootstrap analysis and posterior probabilities ≥ 0.99 in Bayesian phylogenetic inference for the backbone clades of the tree. The inserted tree on the left is one of the most parsimonious trees and indicates branch lengths in MP analysis.

**Figure 2:**
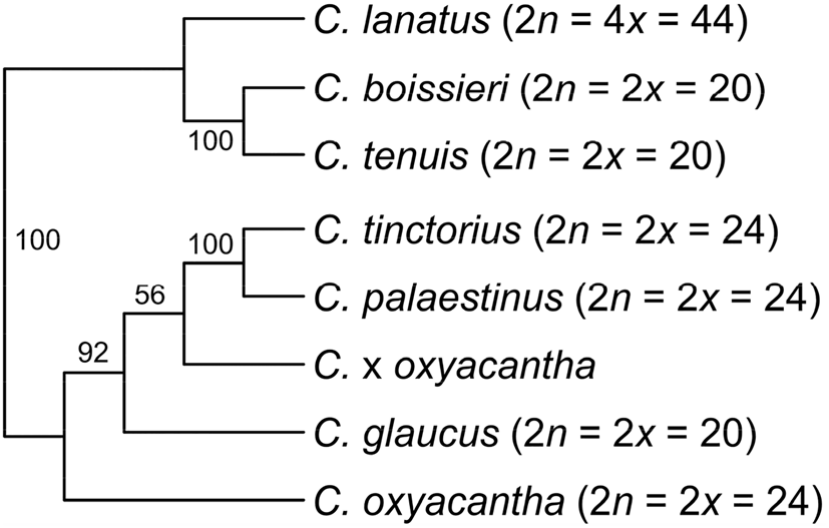
Unrooted SVDquartet species tree using the multi-species coalescent as tree model on the GBS dataset of the *Carthamus* target species. The individuals also analyzed in Fig. 1 were partitioned according to their species affiliation, with the introgressed *C.* × *oxyacantha* individuals treated as a separate category. Ploidy levels and chromosome numbers are listed behind the species names. Numbers along branches indicate bootstrap support values (≥ 50%).

In an initial phylogenetic analysis, we included six *Carthamus* species, i.e. *C. boissieri* halácy, *C. glaucus* m.bieb., *C. lanatus* L. (with three subspecies), *C. oxyacantha*, *C. palaestinus* and *C. tenuis* (boiss. & heldr.) hanelt together with a small but geographically diverse subset of *C. tinctorius* to infer the closest relative of safflower. The GBS-derived data matrix consisted of 108,985 aligned characters of which 3468 were parsimony informative. We used neighbor-joining (NJ), Bayesian phylogenetic inference (BI), maximum parsimony (MP) and SVDquartet (SVDQ) analyses which resulted in identical (BI, MP) or compatible (BI, MP, SVDQ, NJ) tree topologies. The MP analysis retained 2484 equally parsimonious trees with a consistency index (CI) of 0.68 and a retention index (RI) of 0.95. The strict consensus of these trees, summarizing MP (bootstrap, bs) and BI (posterior probabilities, pp) support values, is provided in Fig. 1, the SVDQ tree using the multi-species coalescent as tree model in Fig. 2.

These unrooted trees consist each of two major clades, one including the 24-chromosome species (chromosome numbers according to Vilatersana et al.^3^) *C. oxyacantha*, *C. palaestinus*, and *C. tinctorius* together with a paraphyletic 2*n* = 20 *C. glaucus*, while clade two harbors 2*n* = 20 *C. tenuis* and *C. boissieri*, together with the 2*n* = 44 allotetraploid *C. lanatus*. *Carthamus tinctorius* groups within a paraphyletic *C. palaestinus*, indicating the latter to be the closest relative of safflower and probably the wild progenitor of the crop. The two Iranian *C. oxyacantha* accessions from Isfahan and Kashan are separated from the other members of this species and fall within the *C. palaestinus*/*C. tinctorius* clade (Fig. 1), indicating introgression from these species. Support values for the branches separating the species are generally very high throughout the trees (Figs. 1, 2).

**Figure 3:**
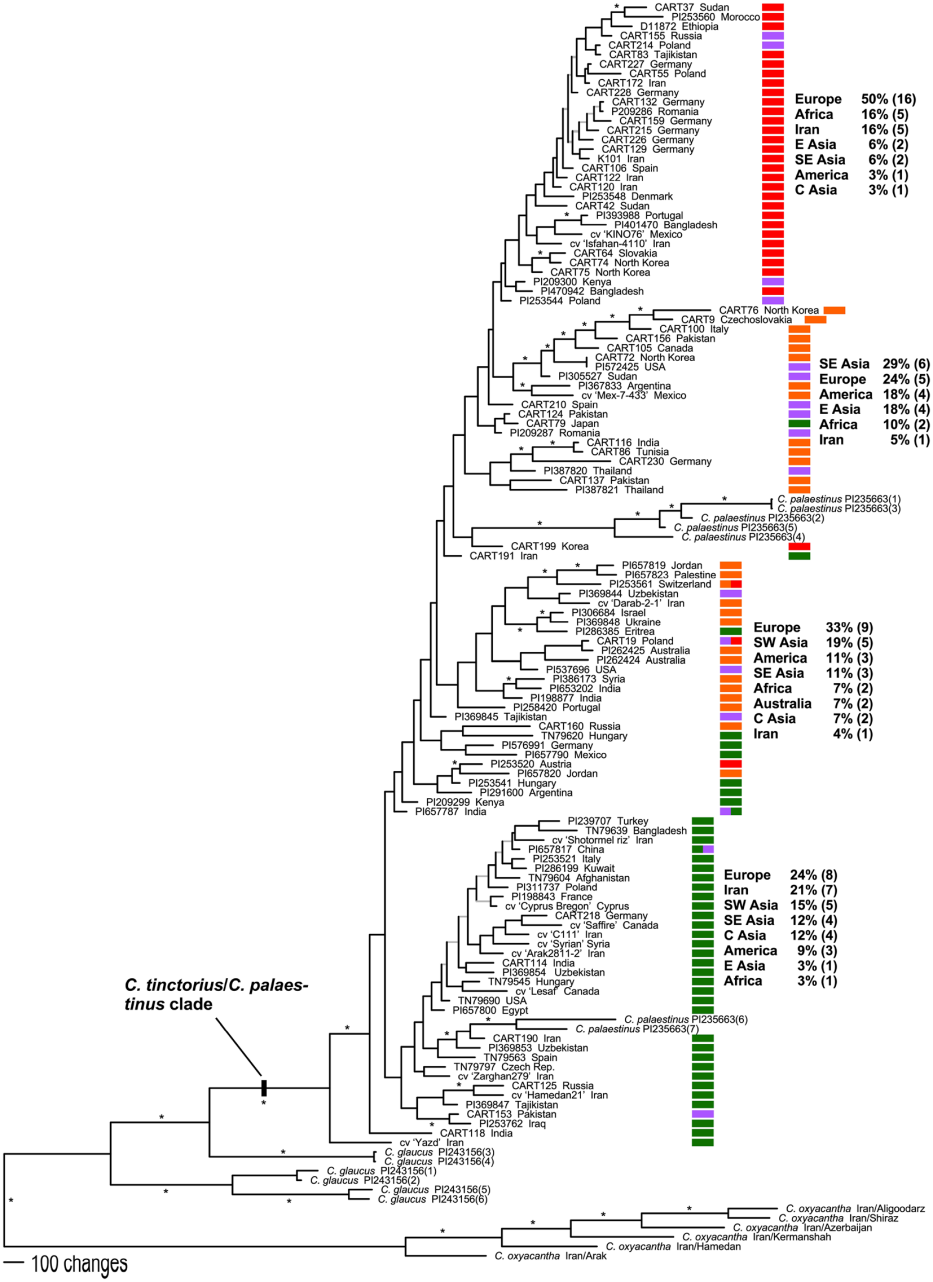
One out of four equally parsimonious trees of a GBS-derived MP analysis of a large and geographically diverse set of 114 safflower accessions. The tree was rooted with *C. oxyacantha.* Bootstrap values ≥ 75% are indicated by asterisks at the branches. Gray branches depict clades that were not recovered in all MP trees and collapse in the strict consensus tree. Colored bars provide the assignment of safflower individuals to four intraspecific groups according to a population structure analysis. To the right of the groups the proportion of members derived from certain geographical areas are given.

Since, *C. tinctorius* was found in a clade with *C. oxyacantha*, *C. palaestinus* and *C. glaucus,* we included these wild species in our analysis of 114 diverse safflower accessions, where we intended to analyze intraspecific groupings within *C. tinctorius*. The alignment for this dataset consisted of 435,024 characters with 7151 of them being parsimony informative. Maximum parsimony analysis of this dataset resulted in four MP trees (CI = 0.30, RI = 0.51) that were rooted with the six non-introgressed *C. oxyacantha* accessions. In this tree (Fig. 3) two clades of *C. palaestinus* group within *C. tinctorius* with 100% bootstrap support for the clade unifying these species. Within *C. tinctorius* bootstrap support values > 75% occur only for few and rather small clades of safflower accessions. The *C. tinctorius* accessions formed four larger groups (Fig. 3), which consisted however of three grades and only one clade. Thus, the borders of these groups and assignment of individuals to them is not unequivocal. We grouped safflower individuals initially into nine geographic regions according to their countries of origin and taking into account earlier proposed groupings^5,10,27,29^. No clear correlation between geographic patterns and phylogenetic groups was found within *C. tinctorius*, i.e. within most groups, safflower accessions from diverse geographical areas were united (Fig. 3). This was also the outcome of a SVDQ analysis for this dataset (not shown).

### Population assignment analysis and Principal Component Analysis (PCA)

**Figure 4:**
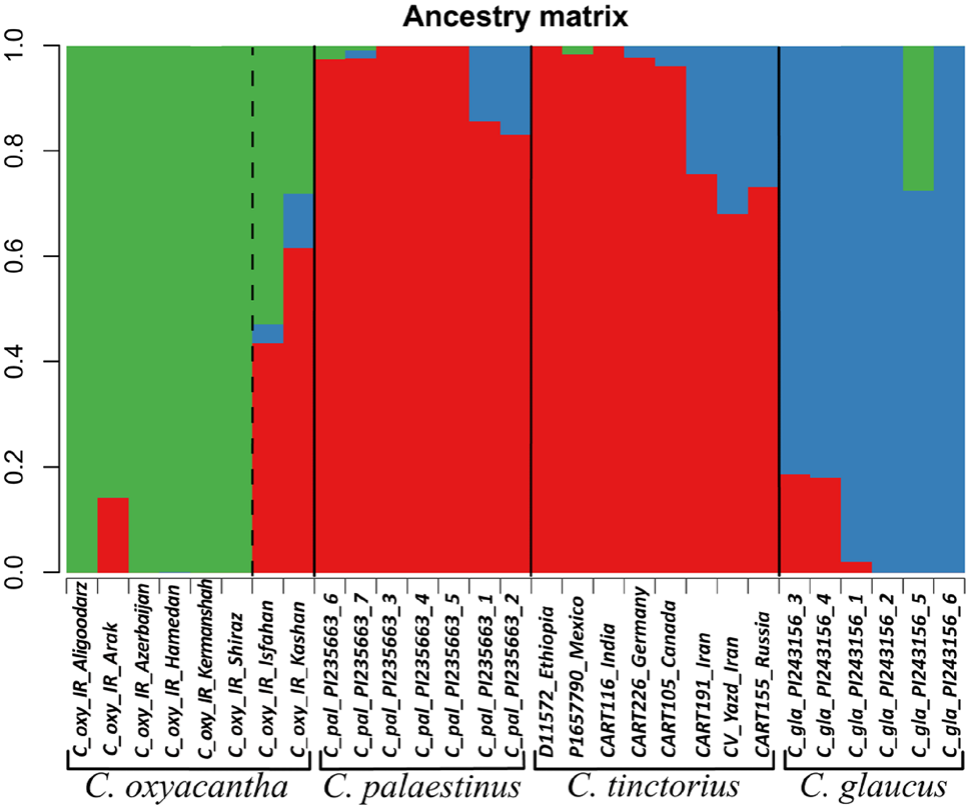
Population structure analysis based on 3720 unlinked SNP loci in LEA with K = 3 for *C. oxyacantha*, *C. palaestinus*, *C. tinctorius* and *C. glaucus*.

**Figure 5:**
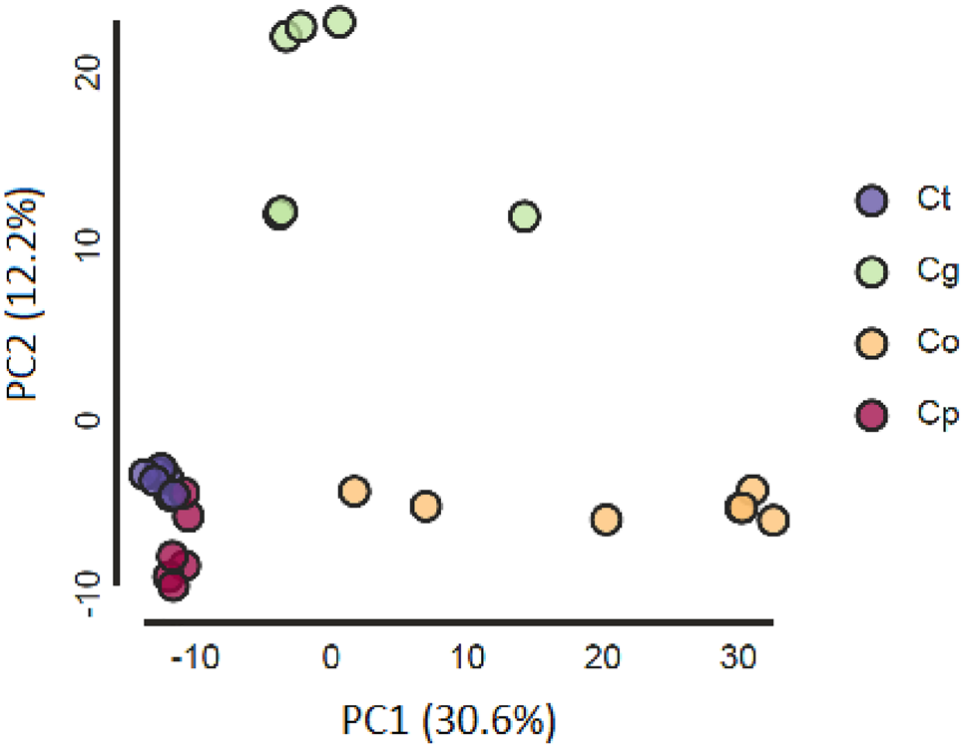
Principal component analysis (PCA) of *C. oxyacantha* (Co), *C. glaucus* (Cg), *C. palaestinus* (Cp) and *C. tinctorius* (Ct) along the first and second PCs.

In addition to tree-based estimations of relationships of *C. tinctorius,* we also used Bayesian population assignment and principal component analyses. *Carthamus oxyacantha*,* C. palaestinus*,* C. glaucus* and *C. tinctorius* individuals were subjected to structure-like analyses in lea. Evanno’s Δk suggested K = 3 as the best-fitting category number that was used for the final analysis (Fig. 4). The analysis confirmed our tree-based assumption that two of the *C. oxyacantha* accessions (from Isfahan and Kashan, Iran) were introgressed by genotypes from the *C. palaestinus*/*C. tinctorius* group and in addition identified also a third accession (from Arak, Iran) showing traces of gene flow. *Carthamus palaestinus* (from Israel) has the greatest similarity with *C. tinctorius* and its gene pool cannot be separated from *C. tinctorius*. Two individuals of *C. palaestinus* (No. 6 and 7) which grouped in tree-based analyses separate from the other five individuals of this species show slight traces of differing alleles (green) in this analysis (Fig. 4). *Carthamus glaucus* (from Lebanon) is clearly distinct from the former two species although some allelic overlap occurs with one Russian and two Iranian safflower accessions (Fig. 4) and two individuals of *C. glaucus* share alleles with *C. palaestinus*/*C. tinctorius*. PCA resulted in three groups of populations (Fig. 5). The first two principal components explained 42.8% of the total molecular variation. PCA results were similar to those of the phylogenetic trees and lea plots, as they grouped *C. tinctorius* and *C. palaestinus* together, while *C. oxyacantha* and *C. glaucus* were clearly separated from them along the first and second axis, respectively.

**Figure 6:**
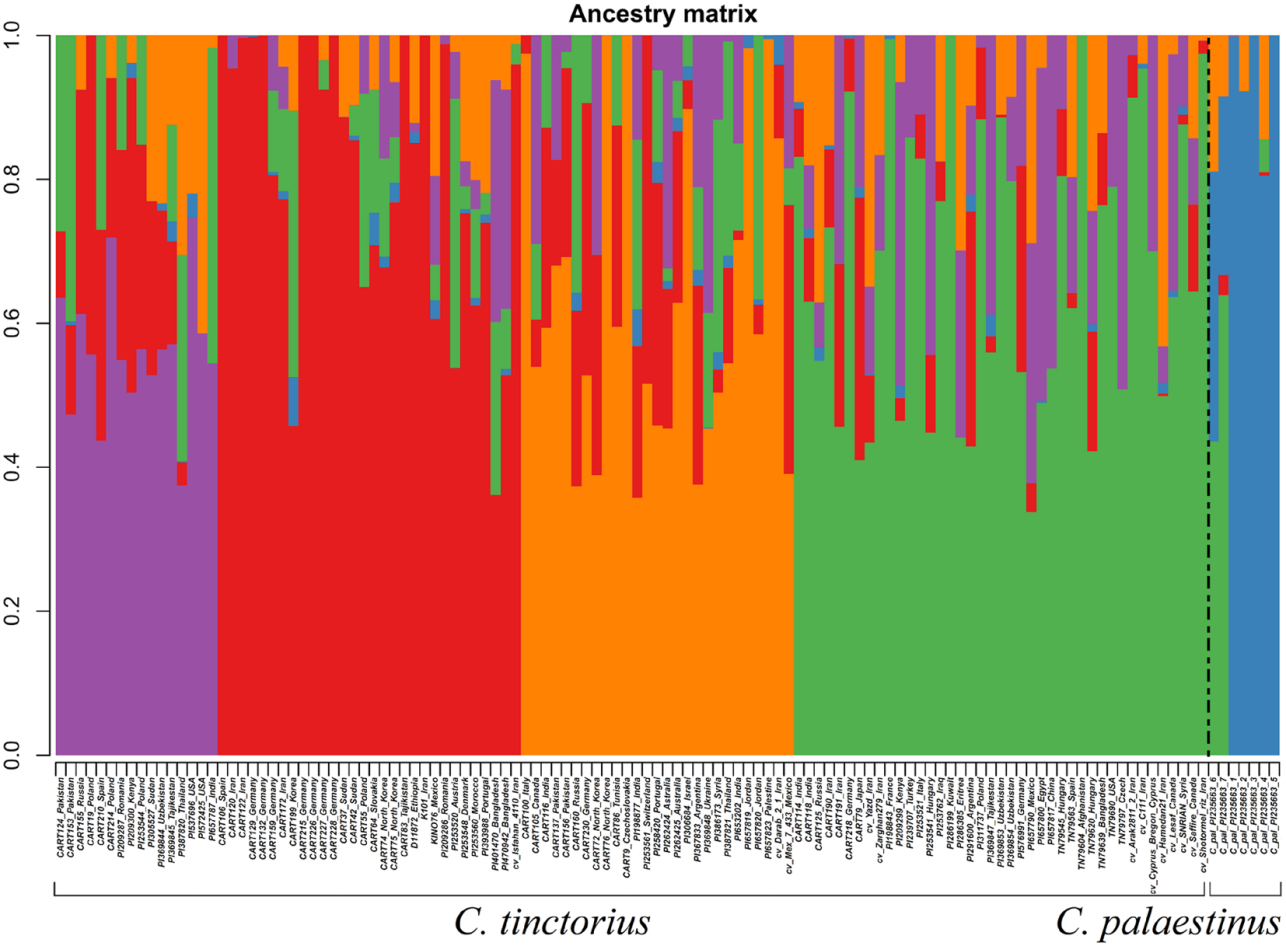
Population structure analysis based on 7556 unlinked SNPs in LEA with K = 5 for *C. tinctorius* and *C. palaestinus* individuals.

To check the likely substructures within the *C. tinctorius* accessions and to gain a more precise understanding of the structural similarities between *C. tinctorius* and its close relative *C. palaestinus*, an additional set of population structure analysis was carried out including only *C. tinctorius* and *C. palaestinus* individuals. For K = 5, four major groups were revealed within *C. tinctorius* samples (Fig. 6). These four groups do not precisely reflect the four groups resulting from the tree-based analysis (Fig. 3) as they are partly mixed in these groups in the tree. However, also based on the population assignment analysis no clear geographic distribution pattern was observed for the major groups and admixture among the four groups is obvious. Most of *C. palaestinus* individuals occur together in a separate group, except for two samples where the majority of the allelic diversity is shared with *C. tinctorius* (Fig. 6).

### Analysis of genome size

Flow cytometry was used to measure genome sizes and infer ploidy levels of *C. tinctorius*, *C. oxyacantha*, and *C. palaestinus* individuals (Table 1)*.* We found that all measured samples of *C. oxyacantha, C. palaestinus* and C. *tinctorius* represent diploid (2*n* = 2*x* = 24) 2C genome sizes ranging from 2.4 to 2.7 pg.

**Table 1:** Genome sizes of *C. tinctorius*, *C. oxyacantha*, and *C. palaestinus* individuals measured by flow cytometry against tomato cv. ‘Stupicke’ (2C = 1.96 pg) as size standard.

Accession	Species	Genome size pg/2C (± SD)
C_tin_TN79548_Hungary	*C. tinctorius*	2.50 (± 0.03)
C_tin_CW74_USA	*C. tinctorius*	2.59 (± 0.05)
C_tin_CART156_Pakistan	*C. tinctorius*	2.49 (± 0.05)
C_tin_CART86_Tunisia	*C. tinctorius*	2.46 (± 0.02)
C_tin_CART118_India	*C. tinctorius*	2.44 (± 0.06)
C_tin_CART227_Germany	*C. tinctorius*	2.50 (± 0.02)
C_tin_K101_Iran	*C. tinctorius*	2.54 (± 0.03)
C_tin_Goldasht_Iran	*C. tinctorius*	2.68 (± 0.09)
C_oxy_IR_Aligoodarz	*C. oxyacantha*	2.54 (± 0.02)
C_oxy_IR_Azerbaijan	*C. oxyacantha*	2.53 (± 0.04)
C_oxy_IR_Hamedan	*C. oxyacantha*	2.40 (± 0.07)
C_oxy_IR_Isfahan	*C.* × *oxyacantha*	2.42 (± 0.05)
C_pal_PI235663_1	*C. palaestinus*	2.60 (± 0.01)

## Discussion

Inferring phylogenetic relationships among closely related species is a challenge because of inter-locus phylogenetic discordance and/or the difficulty of obtaining variable markers. Despite various phylogenetic studies carried out in *Carthamus*, phylogenetic relationships within *Carthamus* are not entirely clear and the closest wild relative of cultivated safflower is still debated due to analyses with limited taxon sampling and/or low information content of the applied markers^2,4,8,22^. Still, studies published during the last decades mostly identified *C. palaestinus* from the southwestern Fertile Crescent as the possible wild ancestor^1–3^, which fits geographically with the earliest archeological remains of safflower that were identified in Syria^11^.

Here we performed phylogenetic and population genomic analyses based on genome-wide distributed SNPs that were obtained through GBS to clarify the phylogenetic relationships of the relatives of *C. tinctorius* and to see if described ecotypes of safflower are genetically distinct. The GBS approach is known to have the potential for better-resolved relationships within and among closely related species in comparison to earlier fragment size analyses or sequence comparisons of single nuclear or chloroplast DNA regions^32–36^.

Our analyses of GBS data representing seven *Carthamus* species resulted in phylogenetic trees (Figs. 1, 2) where *C. boissieri*, *C. lanatus* and *C. tenuis* form one clade that is sister to the clade of *C. glaucus*, *C. oxyacantha*, *C. palaestinus* and *C. tinctorius*. Support values for the clades in the trees are generally very high and the individuals of *C. boissieri*, *C. lanatus*, most *C. oxyacantha*, and *C. tenuis* all form monophyletic groups (Fig. 1). In contrast, *C. tinctorius* individuals group together within *C. palaestinus*, and both together within a paraphyletic *C. glaucus*. Within the *C. palaestinus*/*C. tinctorius* clade also two *C. oxyacantha* accessions grouped, which we assumed to be hybrids between *C. oxyacantha* and *C. palaestinus* or *C. tinctorius*, a result that was confirmed by structure-like analysis. Also in *C. glaucus* two individuals show allelic similarities with *C. tinctorius*, which could indicate gene flow and explains that two clades of a paraphyletic *C. glaucus* were recovered in the phylogenetic trees of which the introgressed individuals are sisters to *C. palaestinus*/*C. tinctorius*. This introgression signal could also explain the reduced support values in the SVDQ tree for the respective clades (Fig. 2). In *C. palaestinus* two individuals group apart from the other members of this species (Figs. 1, 3) and show differences in their allelic patterns (Figs. 4, 6) although they belong to the same accession. It is unclear if this reflects the original diversity of the source population or introgression during ex situ conservation in a gene bank. Our data indicate that hybridization between the species closely related with *C. tinctorius* is still possible on the homoploid level, as the *C. oxyacantha* individual from Isfahan (Iran) was measured with the same genome size as other individuals from this species. The two major clades we obtained in our phylogenetic tree essentially reflect the two sections *Carthamus* and *Atractylis* of Bowles et al.^1^. However, in our analysis *C. glaucus* groups with the section *Carthamus* species while in Bowles et al.^1^ as well as in Vilatersana et al.^3^ and Tarıkahya-Hacıoglu et al.^37^ *C. glaucus* is a member of the sect. *Atractylis* taxa. This inconsistency is however not unique to our analysis. Already in Sasanuma et al.^8^, *C. glaucus* is grouping with *C. tinctorius* while Sehgal et al.^2^ and Mehrotra et al.^9^ retrieved *C. glaucus* accessions in both clades of *Carthamus*. Unfortunately, we relied on gene bank passport data and had no additional seeds available when we obtained the GBS results so we could not check chromosome numbers for our *C. glaucus* individuals to be sure that they are indeed belonging to the 2*n* = 20 chromosomes taxa. As in all before-mentioned studies, *C. glaucus* materials from gene banks were used instead of freshly collected plants from the wild. It might therefore be worthwhile to re-determine gene bank-derived *C. glaucus* accessions in future analyses to be sure about the species affiliation of these lineages.

Intra-species relationships within safflower are not well understood. Based on morphological traits like flower color, inflorescence size, plant height and leaf shape, and their geographical distribution, between five and ten centers of diversity were postulated for safflower, all harboring region-specific ecotypes^1,9,18,19,25^. In our second GBS dataset we included a diverse set of 114 *C. tinctorius* accessions together with the section *Carthamus* species of our initial analysis but excluded the two obviously introgressed *C. oxyacantha* accessions. While *C. glaucus* appears again paraphyletic in the resulting phylogenetic tree (Fig. 3), the individuals of *C. palaestinus* fall within the safflower accessions occurring in two of their different clades. As the *C. palaestinus* individuals in our study show the darker red to brownish flower color (in contrast to the orange flowers of safflower) and seeds are small compared to the crop, we assume that the plants are from a true wild population and do not belong to feral individuals escaping cultivation. This result shows that the gene pool of *C. tinctorius* cannot be separated from that of *C. palaestinus* and is a strong indication that the latter is the wild progenitor of safflower, as its gene pool overlaps with *C. tinctorius*. This result is supported by the population assignment analysis where *C. palaestinus* has the highest similarity with *C. tinctorius* (Fig. 6), and confirms earlier findings regarding the ancestor of safflower^2,12,20^.

Within safflower we found no clear subgroups. *Carthamus tinctorius* in the tree provided in Fig. 3 has four groups of genetically similar accessions. Four groups were also obtained in population assignment analysis (Fig. 6). However, only one of the groups is monophyletic in the phylogenetic tree and none of them shows a clear geographic pattern and none correlates strictly with the groups obtained in the LEA analysis (Fig. 3). Expectedly, bootstrap values within this phylogenetic tree are low, often not even reaching 50%. We interpret this result as an indication of strong trading connections among the early centers of safflower cultivation in Eurasia, followed by multiple independent introductions of safflower germplasm from these centers into regions like the Americas and Australia where safflower cultivation started only during the last century. In addition to the mere introduction of foreign germplasm to new areas also gene flow between newly attained and older local cultivars then further blurred phylogeographic patterns. Admixture among the four different genetic groups of safflower is obvious in most individuals in the population assignment analysis (Fig. 6). This general picture does not change when *C. palaestinus*, *C. oxyacantha* and *C. glaucus* individuals with traces of safflower introgression are removed from the dataset: in this case the resulting clades and subclades in the phylogenetic analysis again lack geographic structure (see Supplementary Fig. S1). This result is in strong contrast to the morphology-defined geographic groups of Ashri^29^, Hanelt^5^, Knowles and Ashri^10^, and Kupcov^27^, which all postulated the presence of certain types typical for specific geographic regions, although they did not arrive at a consensus about the number of such safflower types. This discrepancy can be explained either by the quantitative nature of the used morphological traits that are (i) controlled by very few genetic loci with the respective allelic composition not reflected in the GBS data we obtained and/or (ii) these traits might have an environmental and ecological component that influences characters like flower color or plant height, creating ecotypes where genetic inheritance for the traits might be low. Still, in contrast to our results here Chapman et al.^20^, using 24 nuclear microsatellite markers, found five very weakly supported groups with a possible geographic correlation. In contrast to our dataset, they obtained these groups by restricting their analysis to Ashri’s^29^ set of assumed autochthonous accessions, i.e. a set of materials that were not influenced by introductions during the last century.

For the safflower accessions currently stored in gene banks, we conclude that neither geographic origin nor certain morphological traits are usable indicators of genetic similarity or diversity. For breeding approaches, it is therefore necessary to either use molecular analyses to identify rather diverse genotypes or use gene bank passport data to identify accessions with the preferred or extreme agronomic traits. Moreover, the collections stored at the German National (IPK; identified by “CART” in their accession numbers) and the US Department of Agriculture (USDA; identified by accession numbers starting with “PI”) gene banks seem only marginally overlapping, as two of the phylogenetic groups consist mainly of USDA and two of IPK accessions (Fig. 3). We screened, however, only a part of these collections so that this can also be an artifact of our accession selection.

## Conclusions

Using GBS that combines next-generation sequencing with a genomic complexity reduction approach, phylogenetic relationships of all analyzed safflower accessions could be resolved. However, within *C. tinctorius* we could not recover any geographically defined groups/ecotypes that were postulated earlier based on morphological character combinations thought to prevail in specific regions. This we explain with seed exchange among the areas where safflower is commercially grown, which might have blurred an eventually formerly existing geographic pattern of locally evolved genotypes.

In contrast to the intra-species structure in *C. tinctorius* our GBS analysis recovered species relationships in *C.* sects. *Carthamus* and *Atractylis* with high support values. We identified *C. palaestinus* as closest relative and likely progenitor of safflower. *Carthamus glaucus* resulted as a paraphyletic species that harbors *C. tinctorius*/*C. palaestinus*, but its phylogenetic position in our trees casts some doubts about the species determination of the used material. The other analyzed species appear to be monophyletic.

## Materials and methods

### Taxon sampling

One hundred sixty-seven individuals (see Supplementary Table S1) including 114 cultivated *C. tinctorius* and 53 individuals of wild species of *Carthamus*, i.e. *C. boissieri* (8), *C. glaucus* (6), *C. lanatus* (18), *C. oxyacantha* (8), *C. palaestinus* (7) and *C. tenuis* (6), were included in GBS-based phylogenetic analyses. A set of eight diverse individuals of *C. tinctorius* was used together with individuals of *C. palaestinus*, C. *oxyacantha* and *C. glaucus* for population structure analyses. The analyzed materials were obtained as seeds from the collections of RTIPP-SBUK (Research and Technology Institute of Plant Production, Shahid-Bahonar University, Kerman, Iran), IPK (Leibniz Institute of Plant Genetics and Crop Plant Research, Gatersleben, Germany) and NPGS (National Plant Germplasm System, U.S. Department of Agriculture, USA) and grown at least to a four-leaf stage before harvesting leaves for DNA extraction. We assigned the safflower accessions to nine geographic regions according to their country of origin following roughly the earlier postulated groupings^5,20,29^. These are Southwest Asia (SW Asia: Levant, Turkey, Iraq), Central Asia (C Asia: Kazakhstan, Tajikistan, Uzbekistan), East Asia (E Asia: China, Korea and Japan), Southeast Asia (SE Asia: India and neighboring countries), Europe (including accessions from Russia), Iran (Iran, Afghanistan), Africa, the Americas and Australia. Detailed information such as passport data and observation records for each accession can be found in the online GRIN (Germplasm Resources Information Network) database of NPGS (http://www.ars-grin.gov/npgs/) and Genebank Information System of the IPK Gatersleben (https://gbis.ipk-gatersleben.de/). Collection and use of plant materials was conducted in accordance with institutional, national and international regulations. Seed exchange followed the Nagoya rules of access and benefit sharing through standard material transfer agreements.

### DNA extraction and genotyping-by-sequencing (GBS)

Genomic DNA was extracted from young leaves with a DNeasy Plant Mini kit (QIAGEN). To obtain genome-wide SNPs, we used a two-enzyme GBS protocol that uses the restriction enzymes *Pst*I (NEB Inc.) and *Msp*I (NEB Inc.) to digest 200 ng of genomic DNA. For the library preparation and individual barcoding, the protocol of Wendler et al.^38^ was followed. The individual libraries were sequenced in multiplex on an Illumina HiSeq 2500 (100 bp single-end reads) at the sequencing facilities of Leibniz Institute of Plant Genetics and Crop Plant Research (IPK), Germany targeting a minimum coverage of at least 40×.

### GBS data preparation and filtering

Initial quality assessment of all raw sequence samples was performed using fastqc^39^ to check for overrepresented reads, remaining adapter sequences and to decide on trimming thresholds. Sequences were de-multiplexed allowing for one base mismatch in barcodes, and the restriction site and barcode were trimmed from all GBS sequence reads using cutadapt^40^. Sequence data were quality filtered and clustered using the ipyrad bioinformatics pipeline^41^ with a threshold value for coverage of at least 30×. We conducted two runs of ipyrad resulting in two GBS alignments concatenating all filtered loci: (i) one with a small set of *C. tinctorius* accession together with six wild *Carthamus* species, and (ii) using 114 individuals from *C. tinctorius* plus its closest three relatives according to the prior analysis. The minimal number of samples to possess a certain locus was set to 90% of the individuals. The clustering threshold of reads within and between individuals was set to 0.85 and 0.90 in the first and second datasets, respectively. For the other parameters, the default settings were used. The assembly of the GBS data was done de novo. SNPs were checked for their patterns and found to be nearly completely bi-allelic, even in tetraploid *C. lanatus*. SNP calling could therefore be conducted in ipyrad that has only a setting for diploid taxa.

### Phylogenetic analyses

We used mrbayes 3.2.6^42^ and paup* 4a169^43^ to infer phylogenetic relationships within our set of *Carthamus* species and accessions. NJ, based on General Time Reversible (GTR) distances, MP and SVDquartet trees were calculated in paup* to analyze the aligned sequence data matrices. In MP gaps were treated as missing data and we used a two-step heuristic search with Tree Bisection/Reconnection (TBR) branch swapping as described in Blattner^44^ with initial 500 random addition sequences (RAS) restricting the search to 50 trees per replicate. The resulting trees were afterwards used as starting trees in a search with maxtree set to 10,000. To test clade support, bootstrap analyses were run with re-sampling the dataset 500 times with the same settings as before, except that we did not use the initial RAS step. For both datasets SVDquartets were calculated in paup* running 500 bootstrap re-samples. Individuals were partitioned according to their species affiliation in the smaller dataset including all species while for the dataset with all *C. tinctorius* individuals no partitioning was used. In these analyses trees were selected using Quartet Fiduccia and Mattheyses (QFM) assembly and the multi-species coalescent (MSC) as the tree model. paup* was also used to infer the model of sequence evolution using the Bayesian information criterion.

For BI two times four chains were run for 5 million generations for the dataset including the set of different *Carthamus* species, specifying the model of sequence evolution as GTR + Γ. We sampled a tree every 1000 generations and summarized the trees in mrbayes. Converging log-likelihoods, potential scale reduction factors for each parameter and inspection of tabulated model parameters in mrbayes suggested that stationary had been reached. The first 25% of trees were discarded as burn-in. For the large dataset including all *C. tinctorius* accessions, phylogenetic inference calculations in mrbayes were run with different MCMC settings each for more than three weeks (about 10 million generations) without converging chains when we cancelled these analyses.

### Population structure analyses

The dataset of unlinked SNPs generated from ipyrad was used as input for a Principal Component Analysis (PCA) within ipyrad and for the analysis of population structure for *C. oxyacantha*, *C. palaestinus*, *C. glaucus* and eight *C. tinctorius* cultivars in the R package LEA^45^. The number of subpopulations (K) was tested for a range from one to seven, resulting in an optimal K = 3 for this dataset. An admixture analysis^46^ using sparse nonnegative matrix factorization (snmf) was used for the estimation of population genetic structure. The same analysis approach was used for a dataset including all *C. tinctorius* accessions together with the individuals of *C. palaestinus.* In this case, we arrived at an optimal K = 5.

### Analysis of genome size

To obtain ploidy levels we used flow cytometry to measure the genome sizes of *Carthamus* species^47^. For this, we collected a leaf from each of three individuals from eight *C. tinctorius* and four C*. oxyacantha* accessions and three leaves from one *C. palaestinus* individual. Afterwards, the leaves were transported to the lab and genome sizes were measured on a CyFlow (Sysmex Partec) flow cytometer using propidium iodide (PI) as the staining reagent (Sigma-Aldrich) according to Jakob et al.^48^. The tomato cultivar ‘Stupicke’ was used as the size standard (2C DNA content = 1.96 pg^49^).

## Supplementary Information

Supplementary Information.

## Data Availability

All sequence data are available through the NCBI nucleotide database under bio-project number PRJNA865057.
