# Supplementary material for: Phylogenomic investigation of safflower (Carthamus tinctorius) and related species using genotyping-by-sequencing (GBS)
Source: Sci Rep. 2023 Apr 17;13:6212. doi: 10.1038/s41598-023-33347-0 (PMC10110540; doi:10.1038/s41598-023-33347-0)
Supplement: Supplementary file 1 — Supplementary Information. [file 41598_2023_33347_MOESM1_ESM.pdf]

Supplemental Information

Sardouei-Nasab *et al.*: Phylogenomics of Safflower

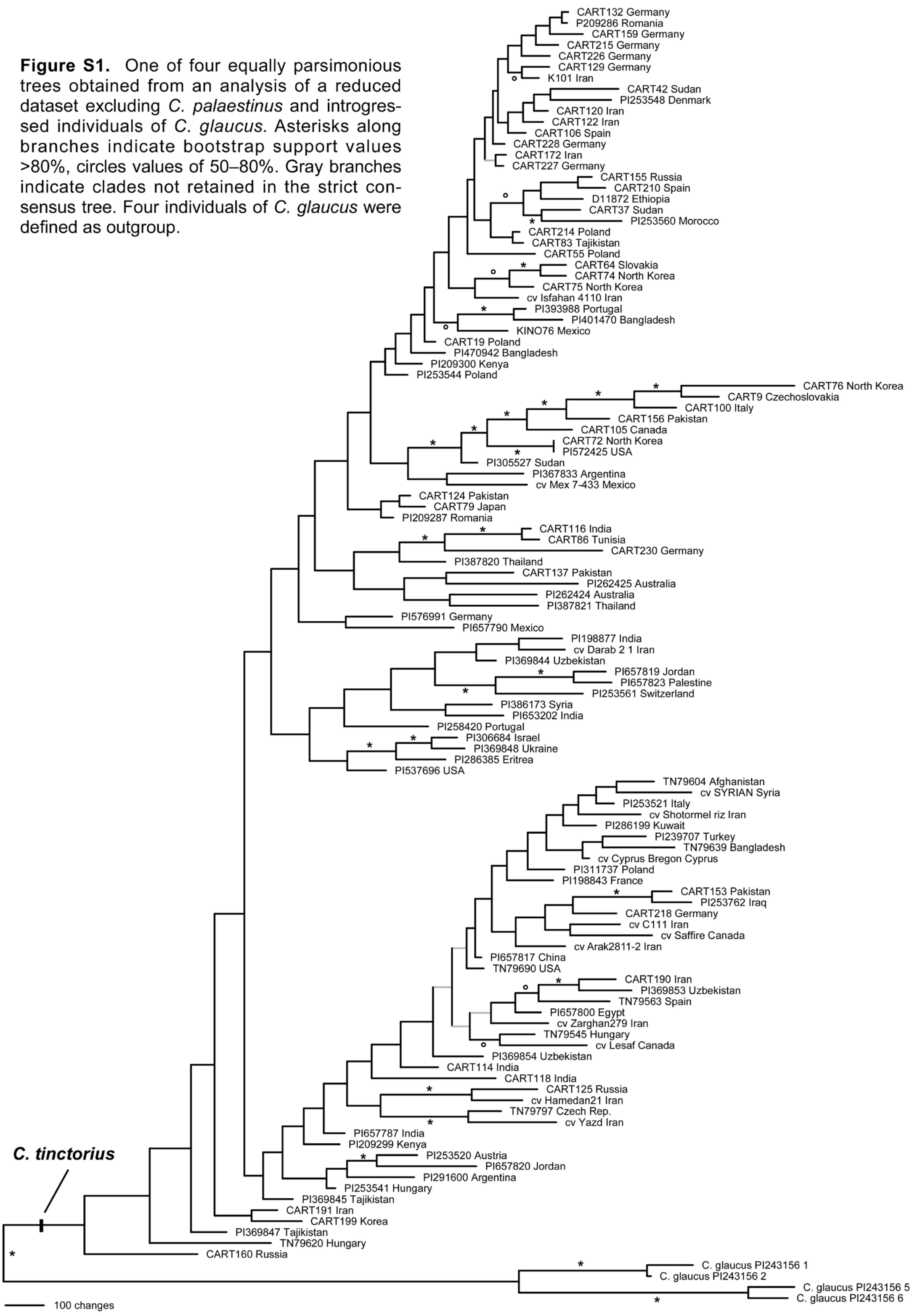

Figure S1. One of four equally parsimonious trees obtained from an analysis of a reduced dataset excluding *C. palaestinus* and introgressed individuals of *C. glaucus.* Asterisks along branches indicate bootstrap support values >80%, circles values of 50–80%. Gray branches indicate clades not retained in the strict consensus tree. Four individuals of *C. glaucus* were defined as outgroup.

Table S1. Studied *Carthamus* L. taxa and their origins.

Species/Name	Accession	Subsp.	Source	Origin	Type
***Carthamus boissieri* Halácsy**					
C_boi_CART85_1	CART85	boissieri	IPK	Greece	Wild
C_boi_CART85_2	CART85	boissieri	IPK	Greece	Wild
C_boi_CART85_3	CART85	boissieri	IPK	Greece	Wild
C_boi_CART85_4	CART85	boissieri	IPK	Greece	Wild
C_boi_CART92_1	CART92	boissieri	IPK	Greece	Wild
C_boi_CART92_2	CART92	boissieri	IPK	Greece	Wild
C_boi_CART92_3	CART92	boissieri	IPK	Greece	Wild
C_boi_CART92_4	CART92	boissieri	IPK	Greece	Wild
***Carthamus glaucus* M.Bieb.**					
C_gla_PI243156_1	PI 243156	glaucus	NPGS	Lebanon	Wild
C_gla_PI243156_2	PI 243156	glaucus	NPGS	Lebanon	Wild
C_gla_PI243156_3	PI 243156	glaucus	NPGS	Lebanon	Wild
C_gla_PI243156_4	PI 243156	glaucus .	NPGS	Lebanon	Wild
C_gla_PI243156_5	PI 243156	glaucus	NPGS	Lebanon	Wild
C_gla_PI243156_6	PI 243156	glaucus	NPGS	Lebanon	Wild
***Carthamus lanatus* L.**					
C_lan_CART50_1	CART50	lanatus	IPK	Bulgaria	Wild
C_lan_CART50_2	CART50	lanatus	IPK	Bulgaria	Wild
C_lan_CART65_1	CART65	lanatus	IPK	Turkmenistan	Wild
C_lan_CART65_2	CART65	lanatus	IPK	Turkmenistan	Wild
C_lan_CART65_3	CART65	lanatus	IPK	Turkmenistan	Wild
C_lan_CART65_4	CART65	lanatus	IPK	Turkmenistan	Wild
C_lan_CART71_2	CART71	turkestanicus (M.Pop.) Hanelt	IPK	Kyrgyzstan	Wild
C_lan_CART71_3	CART71	turkestanicus (M.Pop.) Hanelt	IPK	Kyrgyzstan	Wild
C_lan_CART71_4	CART71	turkestanicus (M.Pop.) Hanelt	IPK	Kyrgyzstan	Wild
C_lan_CART71_5	CART71	turkestanicus (M.Pop.) Hanelt	IPK	Kyrgyzstan	Wild
C_lan_CART73_2	CART73	lanatus	IPK	Italy	Wild
C_lan_CART73_4	CART73	lanatus	IPK	Italy	Wild
C_lan_CART73_5	CART73	lanatus	IPK	Italy	Wild
C_lan_CART82_1	CART82	lanatus	IPK	Georgia	Wild
C_lan_CART82_2	CART82	lanatus	IPK	Georgia	Wild
C_lan_CART82_3	CART82	lanatus	IPK	Georgia	Wild
C_lan_CART84_1	CART84	montanus (Pomel) Jahand. & Maire	IPK	Tunisia	Wild
C_lan_CART93_1	CART93	turkestanicus (M.Pop.) Hanelt	IPK	Uzbekistan	Wild
***Carthamus oxyacantha* M.Bieb.**					
C_oxy_IR_Aligoodarz	Aligoodarz	oxyacantha	RTIPP	Iran	Wild
C_oxy_IR_Arak	Arak	oxyacantha	RTIPP	Iran	Wild

Table S1. Continued

Species/Name	Accession	Subsp.	Source	Origin	Type
C_oxy_IR_Azerbaijan	Azerbaijan sharghi	oxyacantha	RTIPP	Iran	Wild
C_oxy_IR_Hamedan	Hamedan	oxyacantha	RTIPP	Iran	Wild
C_oxy_IR_Isfahan	Isfahan	oxyacantha	RTIPP	Iran	Wild
C_oxy_IR_Kashan	Kashan	oxyacantha	RTIPP	Iran	Wild
C_oxy_IR_Kermanshah	Kermanshah	oxyacantha	RTIPP	Iran	Wild
C_oxy_IR_Shiraz	Shiraz	oxyacantha	RTIPP	Iran	Wild
***Carthamus palaestinus* Eig**					
C_pal_PI235663_1	PI235663	palaestinus	NPGS	Israel	Wild
C_pal_PI235663_2	PI235663	palaestinus	NPGS	Israel	Wild
C_pal_PI235663_3	PI235663	palaestinus	NPGS	Israel	Wild
C_pal_PI235663_4	PI235663	palaestinus	NPGS	Israel	Wild
C_pal_PI235663_5	PI235663	palaestinus	NPGS	Israel	Wild
C_pal_PI235663_6	PI235663	palaestinus	NPGS	Israel	Wild
C_pal_PI235663_7	PI235663	palaestinus	NPGS	Israel	Wild
***Carthamus tenuis* (Boiss. & Blanche) Bornm.**					
C_ten_CART91_1	CART91	foliosus (Boiss.) Hanelt	IPK	Cyprus	Wild
C_ten_CART91_2	CART91	foliosus (Boiss.) Hanelt	IPK	Cyprus	Wild
C_ten_CART91_3	CART91	foliosus (Boiss.) Hanelt	IPK	Cyprus	Wild
C_ten_CART91_4	CART91	foliosus (Boiss.) Hanelt	IPK	Cyprus	Wild
C_ten_CART91_6	CART91	foliosus (Boiss.) Hanelt	IPK	Cyprus	Wild
C_ten_PI244354_1	PI244354	tenuis	NPGS	Israel	Wild
***Carthamus tinctorius* L.**					
CART100_Italy	CART100	tinctorius	IPK	Italy	Cultivated
CART105_Canada	CART105	tinctorius	IPK	Canada	Cultivated
CART106_Spain	CART106	tinctorius	IPK	Spain	Cultivated
CART114_India	CART114	tinctorius	IPK	India	Cultivated
CART116_India	CART116	tinctorius	IPK	India	Cultivated
CART118_India	CART118	tinctorius	IPK	India	Cultivated
CART120_Iran	CART120	tinctorius	IPK	Iran	Cultivated
CART122_Iran	CART122	tinctorius	IPK	Iran	Cultivated
CART124_Pakistan	CART124	tinctorius	IPK	Pakistan	Cultivated
CART125_Russia	CART125	tinctorius	IPK	Russia	Cultivated
CART129_Germany	CART129	tinctorius	IPK	Germany	Cultivated
CART132_Germany	CART132	tinctorius	IPK	Germany	Cultivated
CART137_Pakistan	CART137	tinctorius	IPK	Pakistan	Cultivated
CART153_Pakistan	CART153	tinctorius	IPK	Pakistan	Cultivated
CART155_Russia	CART155	tinctorius	IPK	Russia	Cultivated

Table S1. Continued

Species/Name	Accession	Subsp.	Source	Origin	Type
CART156_Pakistan	CART156	tinctorius	IPK	Pakistan	Cultivated
CART159_Germany	CART159	tinctorius	IPK	Germany	Cultivated
CART160_Russia	CART160	tinctorius	IPK	Russia	Cultivated
CART172_Iran	CART172	tinctorius	IPK	Iran	Cultivated
CART190_Iran	CART190	tinctorius	IPK	Iran	Cultivated
CART191_Iran	CART191	tinctorius	IPK	Iran	Cultivated
CART199_Korea	CART199	tinctorius	IPK	Korea	Cultivated
CART19_Poland	CART19	tinctorius	IPK	Poland	Cultivated
CART210_Spain	CART210	tinctorius	IPK	Spain	Cultivated
CART214_Poland	CART214	tinctorius	IPK	Poland	Cultivated
CART215_Germany	CART215	tinctorius	IPK	Germany	Cultivated
CART218_Germany	CART218	tinctorius	IPK	Germany	Cultivated
CART226_Germany	CART226	tinctorius	IPK	Germany	Cultivated
CART227_Germany	CART227	tinctorius	IPK	Germany	Cultivated
CART228_Germany	CART228	tinctorius	IPK	Germany	Cultivated
CART230_Germany	CART230	tinctorius	IPK	Germany	Cultivated
CART37_Sudan	CART37	tinctorius	IPK	Sudan	Cultivated
CART42_Sudan	CART42	tinctorius	IPK	Sudan	Cultivated
CART55_Poland	CART55	tinctorius	IPK	Poland	Cultivated
CART64_Slovakia	CART64	tinctorius	IPK	Slovakia	Cultivated
CART72_North_Korea	CART72	tinctorius	IPK	North Korea	Cultivated
CART74_North_Korea	CART74	tinctorius	IPK	North Korea	Cultivated
CART75_North_Korea	CART75	tinctorius	IPK	North Korea	Cultivated
CART76_North_Korea	CART76	tinctorius	IPK	North Korea	Cultivated
CART79_Japan	CART79	tinctorius	IPK	Japan	Cultivated
CART83_Tajikistan	CART83	tinctorius	IPK	Tajikistan	Cultivated
CART86_Tunisia	CART86	tinctorius	IPK	Tunisia	Cultivated
CART9_Czechoslovakia	CART9	tinctorius	IPK	Czechoslovakia	Cultivated
D11872_Ethiopia	D11872	tinctorius	RTIPP	Ethiopia	Cultivated
K101_Iran	K101	tinctorius	RTIPP	Iran	Cultivated
KINO76_Mexico	KINO76	tinctorius	RTIPP	Mexico	Cultivated
P209286_Romania	P209286	tinctorius	RTIPP	Romania	Cultivated
PI198843_France	PI198843	tinctorius	RTIPP	France	Cultivated
PI198877_India	PI198877	tinctorius	RTIPP	India	Cultivated
PI209287_Romania	PI209287	tinctorius	RTIPP	Romania	Cultivated
PI209299_Kenya	PI209299	tinctorius	RTIPP	Kenya	Cultivated
PI209300_Kenya	PI209300	tinctorius	RTIPP	Kenya	Cultivated
PI239707_Turkey	PI239707	tinctorius	RTIPP	Turkey	Cultivated
PI253520_Austria	PI253520	tinctorius	RTIPP	Austria	Cultivated

Table S1. Continued

Species/Name	Accession	Subsp.	Source	Origin	Type
PI253521_Italy	PI253521	tinctorius	RTIPP	Italy	Cultivated
PI253541_Hungary	PI253541	tinctorius	RTIPP	Hungary	Cultivated
PI253544_Poland	PI253544	tinctorius	RTIPP	Poland	Cultivated
PI253548_Denmark	PI253548	tinctorius	RTIPP	Denmark	Cultivated
PI253560_Morocco	PI253560	tinctorius	RTIPP	Morocco	Cultivated
PI253561_Switzerland	PI253561	tinctorius	RTIPP	Switzerland	Cultivated
PI253762_Iraq	PI253762	tinctorius	RTIPP	Iraq	Cultivated
PI258420_Portugal	PI258420	tinctorius	RTIPP	Portugal	Cultivated
PI262424_Australia	PI262424	tinctorius	RTIPP	Australia	Cultivated
PI262425_Australia	PI262425	tinctorius	RTIPP	Australia	Cultivated
PI286199_Kuwait	PI286199	tinctorius	RTIPP	Kuwait	Cultivated
PI286385_Eritrea	PI286385	tinctorius	RTIPP	Eritrea	Cultivated
PI291600_Argentina	PI291600	tinctorius	RTIPP	Argentina	Cultivated
PI305527_Sudan	PI305527	tinctorius	RTIPP	Sudan	Cultivated
PI306684_Israel	PI306684	tinctorius	RTIPP	Israel	Cultivated
PI311737_Poland	PI311737	tinctorius	RTIPP	Poland	Cultivated
PI367833_Argentina	PI367833	tinctorius	RTIPP	Argentina	Cultivated
PI369844_Uzbekistan	PI369844	tinctorius	RTIPP	Uzbekistan	Cultivated
PI369845_Tajikistan	PI369845	tinctorius	RTIPP	Tajikistan	Cultivated
PI369847_Tajikistan	PI369847	tinctorius	RTIPP	Tajikistan	Cultivated
PI369848_Ukraine	PI369848	tinctorius	RTIPP	Ukraine	Cultivated
PI369853_Uzbekistan	PI369853	tinctorius	RTIPP	Uzbekistan	Cultivated
PI369854_Uzbekistan	PI369854	tinctorius	RTIPP	Uzbekistan	Cultivated
PI386173_Syria	PI386173	tinctorius	RTIPP	Syria	Cultivated
PI387820_Thailand	PI387820	tinctorius	RTIPP	Thailand	Cultivated
PI387821_Thailand	PI387821	tinctorius	RTIPP	Thailand	Cultivated
PI393988_Portugal	PI393988	tinctorius	RTIPP	Portugal	Cultivated
PI401470_Bangladesh	PI401470	tinctorius	RTIPP	Bangladesh	Cultivated
PI470942_Bangladesh	PI470942	tinctorius	RTIPP	Bangladesh	Cultivated
PI537696_USA	PI537696	tinctorius	RTIPP	USA	Cultivated
PI572425_USA	PI572425	tinctorius	RTIPP	USA	Cultivated
PI576991_Germany	PI576991	tinctorius	RTIPP	Germany	Cultivated
PI653202_India	PI653202	tinctorius	RTIPP	India	Cultivated
PI657787_India	PI657787	tinctorius	RTIPP	India	Cultivated
PI657790_Mexico	PI657790	tinctorius	RTIPP	Mexico	Cultivated
PI657800_Egypt	PI657800	tinctorius	RTIPP	Egypt	Cultivated
PI657817_China	PI657817	tinctorius	RTIPP	China	Cultivated
PI657819_Jordan	PI657819	tinctorius	RTIPP	Jordan	Cultivated

Table S1. Continued

Species/Name	Accession	Subsp.	Source	Origin	Type
PI657820_Jordan	PI657820	tinctorius	RTIPP	Jordan	Cultivated
PI657823_Palestine	PI657823	tinctorius	RTIPP	Palestine	Cultivated
TN79545_Hungary	TN79545	tinctorius	RTIPP	Hungary	Cultivated
TN79563_Spain	TN79563	tinctorius	RTIPP	Spain	Cultivated
TN79604_Afghanistan	TN79604	tinctorius	RTIPP	Afghanistan	Cultivated
TN79620_Hungary	TN79620	tinctorius	RTIPP	Hungary	Cultivated
TN79639_Bangladesh	TN79639	tinctorius	RTIPP	Bangladesh	Cultivated
TN79690_USA	TN79690	tinctorius	RTIPP	USA	Cultivated
TN79797_Czech	TN79797	tinctorius	RTIPP	Czech Rep.	Cultivated
cv_Arak2811_2_Iran	Arak 2811.2	tinctorius	RTIPP	Iran	Cultivated
cv_C111_Iran	C111	tinctorius	RTIPP	Iran	Cultivated
cv_Cyprus_Bregon_Cyprus	cv ‘Cyprus Bre-gon’	tinctorius	RTIPP	Cyprus	Cultivated
cv_Darab_2_1_Iran	Darab 2.1	tinctorius	RTIPP	Iran	Cultivated
cv_Hamedan21_Iran	Hamedan 21	tinctorius	RTIPP	Iran	Cultivated
cv_Isfahan_4110_Iran	Isfahan 4110	tinctorius	RTIPP	Iran	Cultivated
cv_Lesaf_Canada	cv ‘Lesaf’	tinctorius	RTIPP	Canada	Cultivated
cv_Mex_7_433_Mexico	Mex 7-433	tinctorius	RTIPP	Mexico	Cultivated
cv_SYRIAN_Syria	SYRIAN	tinctorius	RTIPP	Syria	Cultivated
cv_Saffire_Canada	cv ‘Saffire’	tinctorius	RTIPP	Canada	Cultivated
cv_Shotormel_riz_Iran	cv ‘Shotormel riz’	tinctorius	RTIPP	Iran	Cultivated
cv_Yazd_Iran	Yazd	tinctorius	RTIPP	Iran	Cultivated
cv_Zarghan279_Iran	Zarghan 279	tinctorius	RTIPP	Iran	Cultivated
